# Myosin activator omecamtiv mecarbil exhibits divergent inotropic and lusitropic effects in cardiac slices from patients with heart failure

**DOI:** 10.1016/j.jmccpl.2023.100041

**Published:** 2023-07-27

**Authors:** Fahimeh Varzideh, Pasquale Mone, Luigi Salemme, Imma Forzano, Angelo Cioppa, Tullio Tesorio, Gaetano Santulli

**Affiliations:** a Department of Medicine, Division of Cardiology, Wilf Family Cardiovascular Research Institute, Einstein Institute for Neuroimmunology and Inflammation (*INI*), Albert Einstein College of Medicine, New York City, NY 10461, USA; b ”Montevergine” Clinic, Mercogliano, Avellino, Italy; c Department of Advanced Biomedical Sciences, “*Federico II*” University, Naples, Italy; d Department of Molecular Pharmacology, Einstein-Mount Sinai Diabetes Research Center (*ES-DRC*), Fleischer Institute for Diabetes and Metabolism (*FIDAM*), Einstein Institute for Aging Research, Albert Einstein College of Medicine, New York City, NY 10461, USA

Over the years, significant efforts have been made to discover new therapeutic agents to improve heart failure (HF) management and patient outcomes. Omecamtiv mecarbil (previously known as CK-1827452), a selective cardiac myosin activator, has recently emerged as a promising treatment option with the potential to enhance cardiac contractility and function.

Omecamtiv mecarbil was developed by Cytokinetics, Inc., in collaboration with Amgen. Its chemical formula is **C**_**20**_**H**_**24**_**FN**_**5**_**O**_**3**_ (the structure is reported in Fig. 1), with a molecular weight of approximately 401.4 g/mol. It is a white to off-white crystalline powder and is usually formulated as an oral tablet or capsule for administration. Omecamtiv mecarbil was identified through a rigorous drug discovery process focused on finding small molecules that could selectively activate cardiac myosin, which is playing a pivotal role in excitation-contraction coupling [1]. Omecamtiv mecarbil displayed promising results in early preclinical studies, leading to further investigations into its therapeutic potential for HF [2].

**Fig. 1:**
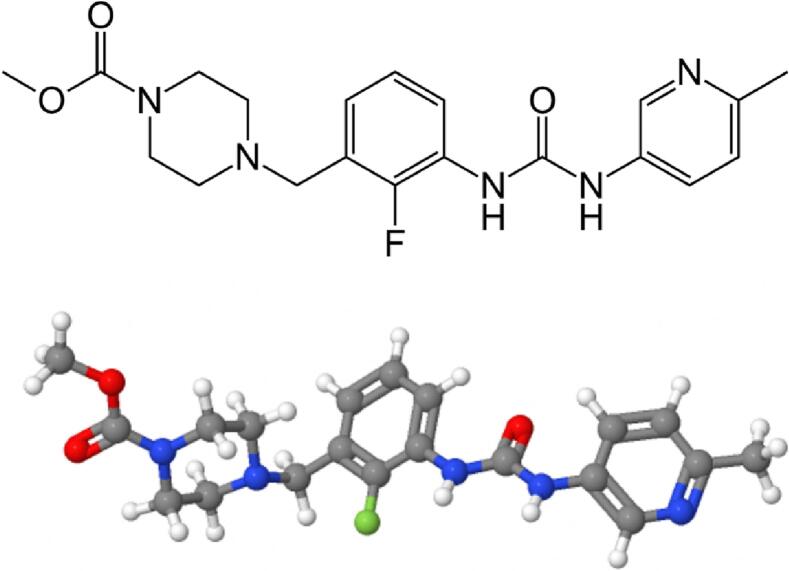
Chemical structure of omecamtiv mecarbil. Skeletal formula (top) and ball-and-stick model (bottom) of omecamtiv mecarbil.

## Mechanism of action

Omecamtiv mecarbil exerts its effects by binding to the cardiac myosin molecule and stabilizing the myosin-ADP complex. This interaction prolongs the attachment of myosin to actin, promoting the transition from a weakly bound to a strongly bound state. By enhancing the rate of actin-myosin cross-bridge formation, omecamtiv mecarbil increases the duration of systolic ejection, leading to improved cardiac contractility without increasing myocardial oxygen consumption [3]. Omecamtiv mecarbil represents an innovative approach to HF treatment, targeting a specific mechanism within the cardiac muscle to improve its function. Its unique chemistry and mechanism of action have garnered significant interest in the medical community and hold promise for potential therapeutic applications in HF management [4].

## From preclinical studies to clinical development

In preclinical studies, omecamtiv mecarbil demonstrated positive effects on cardiac function and performance in animal models of HF [5]. These studies provided valuable insights into its mechanism of action and potential safety profile, paving the way for human clinical trials [6]. The clinical development of omecamtiv mecarbil involved a series of carefully designed clinical trials to assess its safety and efficacy in patients with HF. The first phase 2 double-blind, placebo-controlled, crossover, dose-ranging trial tested the infusion of omecamtiv mecarbil in 45 patients with HF caused by left ventricular dysfunction, showing a significantly improved cardiac function [7].

The ATOMIC-AHF trial (Acute Treatment with Omecamtiv Mecarbil to Increase Contractility in Acute HF) evaluated the short-term effects of intravenous omecamtiv mecarbil in patients hospitalized for acute HF, showing that the treatment did not meet the primary endpoint of dyspnea improvement, but it was generally well tolerated and increased systolic ejection time [8].

The trial COSMIC-HF (Chronic Oral Study of Myosin Activation to Increase Contractility in Heart Failure) revealed that treatment with omecamtiv mecarbil was associated with augmented systolic ejection time, beneficial reverse left ventricular remodeling, improved left ventricular myocardial deformation, a better health-related quality of life, and a reduction in heart rate and N-terminal pro-brain natriuretic peptide (NT-proBNP) concentration in patients with HF with reduced ejection fraction (HFrEF) [9,10].

The subsequent GALACTIC-HF trial (Global Approach to Lowering Adverse Cardiac Outcomes Through Improving Contractility in HF) represents a landmark study that assessed the long-term effects of oral omecamtiv mecarbil on cardiovascular outcomes in a large population of patients with chronic HF [11,12]. The GALACTIC-HF trial was designed to assess whether treatment with omecamtiv mecarbil could improve cardiovascular outcomes and reduce HF-related hospitalizations and mortality. The GALACTIC-HF trial enrolled a large and diverse population of HF patients. Patients were randomly assigned to receive either omecamtiv mecarbil or placebo in addition to their standard HF medications [11,12]. The trial was a multicenter, international study conducted at numerous clinical sites worldwide. It was designed as a double-blind, placebo-controlled trial to ensure the reliability of the results and reduce potential bias. Patients enrolled in the trial underwent a comprehensive evaluation of their HF status, medical history, and baseline characteristics before randomization. Patients in the treatment group received omecamtiv mecarbil orally, in addition to their standard HF medications. The dosing regimen and titration were warily controlled to achieve the desired therapeutic effect while minimizing potential adverse effects. Subjects were then followed up to assess the effects of omecamtiv mecarbil on HF-related events and overall cardiovascular outcomes. The primary endpoint was a composite of first HF event or death from cardiovascular causes. Secondary endpoints included changes in health-related quality of life, exercise tolerance, death from cardiovascular causes, and other cardiovascular outcomes. Omecamtiv mecarbil improved cardiac function and reduced HF symptoms, leading to increased systolic ejection time and stroke volume. The GALACTIC-HF trial revealed a reduction in HF-related hospitalizations and cardiovascular death in the omecamtiv mecarbil-treated group, demonstrating its potential to positively impact HF management. However, the secondary outcomes of death from cardiovascular causes, first hospitalization for HF, and change in the total symptom score on the Kansas City Cardiomyopathy Questionnaire, did not reach a statistically significant difference between groups.

Exercise limitation is a cardinal manifestation of HFrEF. A dedicated trial was designed to specifically address this aspect: Study to Assess the Effect of Omecamtiv Mecarbil on Exercise Capacity in Subjects with HF (METEORIC-HF). This trial evidenced that in HFrEF patients omecamtiv mecarbil did not significantly improve exercise capacity over 20 weeks compared with placebo; these negative results do not support the use of omecamtiv mecarbil for treatment of HFrEF for improvement of exercise capacity [13].

The exact molecular mechanisms underlying the different outcomes evidenced by these trials remain to be fully understood. For instance, some aspects of the effects of omecamtiv mecarbil on cardiomyocyte contraction and relaxation remain to be defined [[14], [15], [16]]. In this sense, in a timely study published in the current issue of *JMCC Plus*, Amesz and collaborators used living myocardial slices from patients with HF to evaluate the direct biomechanical effects of omecamtiv mecarbil as compared to dobutamine [17]. Living myocardial slices present an innovative platform for pre-clinical experimentation with novel HF drugs. Specifically, the slices for this study were obtained from patients with end-stage HF undergoing cardiac transplantation or left ventricular assist device implantation; the slices were cultured under electromechanical stimulation (diastolic preload: ∼1 mN, stimulation frequency: 0.5 Hz). The treatment with omecamtiv mecarbil significantly increases contractile force and time-to-peak of living myocardial slices from patients with HF. These results are in agreement with another recent report showing that omecamtiv mecarbil augments cardiomyocyte contractile activity both at resting and systolic Ca^2+^ levels [18].

However, omecamtiv mecarbil also slows relaxation, which could lead to diastolic filling abnormalities. Remarkably, the slowed relaxation of living HF slices induced by omecamtiv mecarbil is further aggravated at faster heart rates. These data strongly suggest a limited application of omecamtiv mecarbil in HF condition with a prevalent diastolic dysfunction, like diabetic cardiomyopathy and HF with preserved ejection fraction (HFpEF).

The main limitations of the study include the limited number of patients (five), not having accounted for differences in HF drugs received by the patients, and the lack of control groups like comparison with vehicle-treated HF specimens as well as testing slices from non-HF subjects.

In summary, omecamtiv mecarbil represents a novel approach to the treatment of HFrEF by directly targeting cardiac contractility. It may therefore complement existing HF therapies, providing additional benefits to patients with impaired cardiac function and potentially reducing the burden of hospitalizations and mortality associated with the condition. Omecamtiv mecarbil has rapidly emerged as a promising cardiac medication, offering a new mechanism of action to improve cardiac contractility in HF patients. Its history and development have been marked by significant progress from preclinical studies to pivotal clinical trials. As the field of HF research continues to evolve [19,20], omecamtiv mecarbil holds promise as a potentially transformative treatment option for the management of HFrEF.

## Declaration of competing interest

None.
